# Supplementary material for: MetaRibo-Seq measures translation in microbiomes
Source: Nat Commun. 2020 Jun 29;11:3268. doi: 10.1038/s41467-020-17081-z (PMC7324362; doi:10.1038/s41467-020-17081-z)
Supplement: Supplementary file 10 — Supplementary Data 7 [file 41467_2020_17081_MOESM10_ESM.zip › File2/Confidence_High_Taxonomy/291496_out.krona.html]

Javascript must be enabled to view this page.

members
magnitude
magnitudeUnassigned
count
unassigned
taxon
rank

291496\_out

3

superkingdom
2
3

phylum
976
3

class
200643
3

3
171549
order

3
2005525
family

genus
3
375288


SRS012849\_contig\_number\_26296SRS055017\_contig\_number\_17549SRS893366\_contig\_number\_18926
species
1750560
3
